# Genetic and Epigenetic Consequence of Early-Life Social Stress on Depression: Role of Serotonin-Associated Genes

**DOI:** 10.3389/fgene.2020.601868

**Published:** 2021-01-22

**Authors:** Tomoko Soga, Chuin Hau Teo, Ishwar Parhar

**Affiliations:** Brain Research Institute, Jeffrey Cheah School of Medicine and Health Sciences, Monash University Malaysia, Bandar Sunway, Malaysia

**Keywords:** serotonin, depression, social stress, epigenetic modification, 5-HT receptor

## Abstract

Early-life adversity caused by poor social bonding and deprived maternal care is known to affect mental wellbeing and physical health. It is a form of chronic social stress that persists because of a negative environment, and the consequences are long-lasting on mental health. The presence of social stress during early life can have an epigenetic effect on the body, possibly resulting in many complex mental disorders, including depression in later life. Here, we review the evidence for early-life social stress-induced epigenetic changes that modulate juvenile and adult social behavior (depression and anxiety). This review has a particular emphasis on the interaction between early-life social stress and genetic variation of serotonin associate genes including the serotonin transporter gene (5-HTT; also known as SLC6A4), which are key molecules involved in depression.

## Introduction

History of early-life social stress indicates adverse effects on functions of the hypothalamic-pituitary-adrenal axis and stress response in later life (Denhardt, 2018; Lapp et al., 2019) linked to the development of the major depressive disorder in adolescents and adults (Hettema et al., 2006; Pace et al., 2006; Rao et al., 2008; Heim and Binder, 2012; Bunea et al., 2017). These clinical findings are backed by animal studies demonstrating that poor social bonding and reduced maternal care can subsequently cause altered behavior and heightened anxiety, as well as negative consequences on the brain development of offsprings (Eiland and McEwen, 2012; Carini and Nephew, 2013; Murgatroyd et al., 2015).

The effect of early-life social stress on the genetics of depression can be described as the influence of the environment on the genes of the brain – in other words, epigenetics. Epigenetics involves modifications to gene expression that may be inherited by the offspring, without any changes in the DNA sequences that encodes for those genes (Hochberg et al., 2011). Epigenetic modifications involve three processes, DNA methylation, histone modification, and various RNA-mediated processes. In DNA methylation, a methyl group is transferred to C-5 of a cytosine residue in DNA – this interferes with the ability of transcription factors to bind to DNA, and as such, high methylation levels are associated with repression of gene expression (Crabtree, 2020). Histone modification, on the other hand, involves either methylation, acetylation, or phosphorylation of amino acids in the histone protein tails; as the histones control how tightly chromatin is coiled and a tightly packed chromatin restricts access of regulatory factors to DNA, modification of those histones can control how much genes are expressed (Crabtree, 2020). Finally, non-coding RNAs can facilitate chromatin modifications, while microRNAs can pair to complementary target mRNAs, directly suppressing translation from mRNA to protein (Crabtree, 2020).

It is well known that early-life social stress leads to persistent epigenetic modifications of target genes associated with changes in emotional behavior (Nugent et al., 2011; McCann et al., 2017; Fogelman and Canli, 2019). There has been a growing body of work in the past decade, documenting epigenetic action in the brain, stemming from exposure to early-life social stress in animal models and human studies (Murgatroyd et al., 2009; McClelland et al., 2011; Heim and Binder, 2012; Huang, 2014; Provençal and Binder, 2015; Vaiserman, 2015). These studies have indicated various lasting changes in gene expression due to early-life stress, such as altered arginine vasopressin expression (Murgatroyd et al., 2009) and increases in seizure and epilepsy incidences (Huang, 2014). Prenatal stress caused elevated methylation of a glucocorticoid receptor gene in infants, altering their reactivity to stress (Oberlander et al., 2008). A genome-wide study discovered significant methylation differences within promoters of subjects exposed to early-life stress; 248 promoters showed hypermethylation, while 114 showed hypomethylation. The expressions of genes involved in neuronal plasticity, in particular, were significantly different (Labonté et al., 2012). Genes undergoing methylation in association with early-life social stress-induced depression are also well studied (Schoenherr and Anderson, 1995; Jiang et al., 2019). The monoaminergic theory is supported as the main neuropathogenesis of depression. Based on this, epigenetic modifications of monoamine-related genes such as transporters, metabolic enzymes, synthesis enzymes, and receptors have been well investigated to understand the neuropathogenesis of depression.

Serotonin (5-hydroxytryptamine, 5-HT) is the main monoamine system involved in the neuropathogenesis of depression. The key 5-HT-related genes are serotonin transporter (5-HTT; also known as SLC6A4), monoamine oxidase A (MAO-A), tryptophan hydroxylase 2 (TPH2), and 5-HT receptors. These 5-HT-related genes and their signaling pathways are involved in brain development, stress response, and emotional control. Epigenetic alterations of 5-HT-related genes may be the underlying effect of early life stress on depression (Parade et al., 2017). Since depression is closely tied to the 5-HT system, therefore, the examination of epigenetic influence on 5-HT-associated genes could generate an interesting body of work that would serve better to explain the interplay between nature and nurture in depression. In this review, we looked at the mounting evidence for early life social stress-induced epigenetic changes in several 5-HT-associated genes and how these gene modifications influence behavior in later life. Besides, treatments that reduce early-life social stress are also reviewed to understand their impact on the attenuation of genetic and behavioral changes.

## Early Life Social Stress-Induced Epigenetic Changes in Serotonin-Related Genes

### Serotonin Transporter

The 5-HTT gene was first sequenced and characterized by Lesch et al. (1994), but its function in terminating serotonergic neurotransmission was already documented earlier (Kanner and Schuldiner, 1987). Variants of 5-HTT gene potentially increase susceptibility to a stressful environment, increasing the risk for mental disorders. There have been numerous studies and reviews covering the role of 5-HTT in psychiatric disorders, indicating that polymorphisms in the 5-HTT lead to serotonergic dysfunction that can develop into various diseases such as major depressive disorder and bipolar disorder (Mann et al., 2000; Zanardi et al., 2000; Hahn and Blakely, 2002; Anguelova et al., 2003; Murphy et al., 2004; Abdolmaleky et al., 2014). An epidemiology study performed on a cohort of 1,037 children identified a correlation between the presence of a functional polymorphism in the 5-HTT gene in individuals having a heterozygous or homozygous variant of the short allele, who exhibited greater susceptibility to life stress history for predicting depression, compared to individuals with a homozygous long allele variant (Caspi et al., 2003).

The influence of epigenetics on 5-HTT is also well documented. For example, DNA hypermethylation, an indicator of epigenetic influence, has been observed in the 5-HTT gene of schizophrenic patients alongside reduced 5-HTT expression (Abdolmaleky et al., 2014). Increased methylation in the proximal promoter region of 5-HTT is an epigenetic change that has a positive correlation with increased responsiveness to threat in the amygdala (Nikolova et al., 2014).

As stress is one of the major drivers for epigenetic changes in the brain (Gudsnuk and Champagne, 2012), it stands to reason that early life social stress would also have a major effect on epigenetic modifications to the 5-HTT gene. Methylation of retro-transposonal AluJb element is associated with stress response under major depressive disorder, where lower methylation has a better stress-adaptive reaction (Schneider et al., 2018). The link between methylation and depression is influenced by genetic variation – specific genotypes have higher methylation associated with depression, while those homozygous for short 5-HTT alleles exhibit lower methylation in association with depression (Lam et al., 2018). DNA methylation of 5-HTT in the 10-year-old twins experiencing discordant stress through bullying has been demonstrated to be significantly higher compared to their co-twin that did not undergo bullying in the same period (Ouellet-Morin et al., 2013). The focus on twins indicates that childhood bullying – early life social stress – is the influencer for increased 5-HTT methylation rather than predetermined genetic factors. The bullied twin also exhibited blunted cortisol responses in comparison to the non-bullied twin (Ouellet-Morin et al., 2013).

Research using animal models supports the role of stress during early life – it causes epigenetic changes that may be associated with risk of depression. Peer rearing in rhesus macaques, which is a form of early life stress in comparison to maternal rearing (Harlow and Suomi, 1974; Suomi et al., 1976), causes reduced H3K4me3 (Histone 3 protein with trimethylation at lysine 4) binding at the promoter of the 5-HTT gene (Lindell et al., 2012). As H3K4me3 is an epigenetic modification that promotes gene expression, lower H3K4me3 indicates lower 5-HTT expression. This is further supported by the finding of serotonin metabolite 5-HIAA in the cerebrospinal fluid of peer-reared macaques, which suggests a decreased serotonergic function in the central nervous system (Lindell et al., 2012).

Homozygous and heterozygous 5-HTT knockout rats when exposed to early-life stress show decreased serotonergic innervation to Edinger-Westphal urocortin 1 neurons (van der Doelen et al., 2017). Abnormal levels of urocortin 1 have been associated with major depressive disorder (Ryabinin et al., 2012; Waters et al., 2015), suggesting that early-life stress can interact with 5-HTT to cause depressive-like neurophysiology. Furthermore, heterozygous 5-HTT knockout rats exposed to early life social stress triggered by maternal separation, exhibit anhedonic behavior in the form of lower sucrose preference (Houwing et al., 2019). The same study also found low gene expression of nerve growth factor. Examination of clinical studies has reported a significant correlation between reduced nerve growth factor expression and the diagnosis of major depressive disorder (Chen et al., 2015). As a whole, these studies provide strong support for the role of epigenetic action on 5-HTT in early life social stress.

### Monoamine Oxidase A

MAOA is involved in breaking down serotonin. An increase in MAOA expression results in a decrease in serotonin levels in the brain, which has been suggested as the main factor in major depressive disorder (Naoi et al., 2018). Epigenetic regulation of MAOA has been documented in humans (Shumay et al., 2012). In particular, methylation of MAOA in the promoter region of CpG5 and CpG11 increases MAOA expression, which in turn decreases serotonin levels; this has been observed in female patients with depression (Domschke et al., 2015). Behavioral disinhibition in children has been associated with a functional promoter polymorphism on *MAOA* (*MAOA-LPR*) that interacts with early life social stress (Enoch et al., 2010). Furthermore, while early life social stress has been associated with increased aggressive disorders in males through the *MAOA-L* allele, such stress exposes *MAOA-L* females to a higher risk of developing depression (Melas et al., 2013). It has been suggested that the susceptibility of *MAOA-L* females to depression may be a result of epigenetic dysregulation of *MAOA* by early life stressors, which affects DNA methylation of the glucocorticoid receptor gene *NR3C1* (Melas et al., 2013).

Studies in rodents have also drawn similar connections between early life stress and epigenetic control of MAOA. Early life social stress induces CpG-specific methylation in the *MAOA* promoter, which elevates MAOA expression in the dorsal striatum – this is associated with voluntary alcohol consumption (Bendre et al., 2019). The effect of peripubertal stress on the epigenetic state of *MAOA* is associated with the development of antisocial behavior (Márquez et al., 2013). The development of aggressive behavior is sexually dimorphic, with MAOA hypermethylation in the hypothalamus and in the prefrontal cortex of male rats. In contrast, female rats do not exhibit any changes in epigenetic control of *MAOA* (Konar et al., 2019).

### Tryptophan Hydroxylase 2

The gene for tryptophan hydroxylase 2 (TPH2) is a neuron-specific rate-limiting 5-HT biosynthetic enzyme in the brain. Alterations in *TPH2* gene expression is involved in the pathogenesis and treatment of MDD (Tsai et al., 2009; Xu et al., 2012). Single-nucleotide polymorphisms (SNPs) in *TPH2* gene are linked to 5-HT dysfunction (Gao et al., 2012), which have been associated with MDD (Zill et al., 2004; Zhang et al., 2005), and one of the SNPs in the TPH2 gene is associated with amygdala and hippocampal volume (Inoue et al., 2010). The promoter region of the *TPH2* gene lacks a CpG island; however, there are numerous scattered CpG sites and an enriched signal of DNA hypomethylation at the 5′-UTR locus (Chen and Miller, 2012). A recent study has shown that hypermethylation of the CpG-site in the *TPH2* gene during early-life stress could reduce antidepressant response within the first 2 weeks of treatment in patients with MDD (Xu et al., 2016; Shen et al., 2020). Furthermore, methylation of a single CpG site in the promoter region of TPH2 significantly decreases *TPH2* gene expression levels. This methylation is also partially linked with suicide in MDD patients (Zhang et al., 2015). These studies suggest the impact of early life social stress-associated epigenetic action on *TPH2* gene and depression.

### 5-HT Receptors

Seven families of 5-HT receptors and their subtypes have been identified, namely, 5-HTI (5-HT1A, 5-HT1B, 5-HTID, 5-HTIE, and 5-HT1F), 5-HT2 (5-HT2A, 5-HT2B, and 5-HT2C), 5-HT3, 5-HT4, 5-HT5 (5-HT5A and 5-HT5B), 5-HT6, and 5-HT7. 5-HT receptor-specific agonists and antagonists have been designed and developed as therapeutics against mental disorders. Among the 5-HT receptors, the most well-studied receptor is 5-HT1A, known as an autoreceptor, which has inhibitory control over the 5-HT neuronal activity. Increased levels of 5-HT1A in 5-HT neurons of the dorsal raphe have been reported in MDD patients and suicide victims with MDD (Stockmeier et al., 1998; Hesselgrave and Parsey, 2013). 5HT1A is also a postsynaptic receptor, expressed in main target brain areas, the hippocampal, cortical, and hypothalamic regions, that are associated with depression, stress, and anxiety (Albert et al., 2019). Several SNPs and stress-induced DNA methylation of the 5-HT1A promoter have been associated with MDD and alteration in their response to antidepressants. C(−1,019)G (rs6295) is a functional 5-HT1A promoter polymorphism that modifies *5-HT1A* gene expression in a brain region-specific manner (Le Francois et al., 2008) and modify connectivity such as amygdala-ventrolateral prefrontal cortex, and corticolimbic connectivity related to MDD (Vai et al., 2017). In fact, C(−1,019)G (rs6295) promoter polymorphism in 5-HT1A elevated risk of depression (Benedetti et al., 2011; Kim et al., 2011; Vai et al., 2017), resistance to an antidepressant (Wang et al., 2018b), panic disorder (Choe et al., 2013), fear (Straube et al., 2014), gender-dependent modulatory effects on depression, physical function in patients with pain (Lebe et al., 2013), and suicidal attempt in MDD (Sawiniec et al., 2007). Kim and co-workers have reported interactions between C-1019G polymorphism in 5HT1A and negative life stressors that account for MDD symptoms (Kim et al., 2011). These findings support those genetic alterations of the 5-HT1A promoter that make it sensitive to stress and increase the risk of MDD.

Some studies have suggested that human *5HT1A* gene methylation is associated with MDD. Increased DNA methylation of 5HT1A promoter in leukocytes has been reported in bipolar depression (Carrard et al., 2011). Stress-linked hypomethylation of CpG668 site in the *5HT1A* gene from blood samples is associated with resistance to antidepressants in treatment-naive MDD patients (Wang et al., 2018a).

Studies in animal models suggest that early-life social stress induces persistent changes in 5-HT1A expression levels in the amygdala, hippocampus, and dorsal raphe nucleus (Bravo et al., 2014). Furthermore, early-life stress, in combination with adult social isolation, dramatically decreases the 5-HT1A-mediated inhibition of layer II/III pyramidal neuronal activities (Goodfellow et al., 2009). Le Francois and co-workers have reported methylation of 24 CpG sites on the mouse 5-HT1A promoter, and chronic mild stress increased DNA methylation of a single site located within the Sp4 element of the *5HT1A* gene that correlates with increased mRNA expression levels in the raphe and prefrontal cortex in male mice (Le Francois et al., 2015). In brief, subjects with methylation of the *5-HT1A* gene variant may be more susceptible to developing MDD.

Other variants of 5-HT receptors have also been reported to have a risk of MDD. Methylation of 5-HTR2A genotype at two CpG sites (−1,420 and −1,224) has been associated with PTSD and MDD under contextual stress (Parade et al., 2017). An SNP in the allele of −1438A/G (rs6311) in the 5HTR2A promoter is highly influenced by genetic factors and the environment in female MDD patients (Lebe et al., 2013). These studies support that 5HT2A methylation is a mechanism by which early adversity is biologically encoded. In another case, epigenetic modification of the 5-HT3A is involved in the molecular mechanism underlying the relationship between childhood maltreatment and the severity of neuropsychiatric diseases in adulthood (Perroud et al., 2016). These studies of epigenetic regulation of 5-HT and 5-HT receptors could be applied for more effective personalized treatments for MDD.

### Brain-Derived Neurotrophic Factor

Brain-derived neurotrophic factor (BDNF) is a neurotrophin involved in many of the brain’s activities, including, but not limited to, neuronal development, synaptic modulation, and plasticity, as well as hippocampal function (McAllister et al., 1999; Huang and Reichardt, 2001; Lu, 2003; Monteggia et al., 2004). While BDNF plays a role in serotonergic expression, it may also itself be regulated by 5-HT, particularly in depression and stress (Martinowich and Lu, 2008). During the depression, the role of BDNF can vary; in the hippocampus and the prefrontal cortex, BDNF expression is associated with inhibition of depressive symptoms, whereas it promotes anxiety-like symptoms in the nucleus accumbens and the amygdala (Yu and Chen, 2011). Higher DNA methylation of the *Bdnf* gene has been associated with the improved antidepressant response, with escitalopram treatment increasing methylation after 8 weeks (Wang et al., 2018b).

The effect of early life stress on BDNF has been well studied in the past decade. Maltreatment of rat pups by stressed caretakers during infancy elicited significant methylation of *Bdnf* exons in the prefrontal cortex – the presence of methylation persists even into adulthood, demonstrating a long-term effect (Roth et al., 2009). The postnatal maternal separation was found to induce a decrease in exon IV *Bdnf* mRNA, with adult restraint stress further exacerbating the maternal separation-induced drop in BDNF expression (Seo et al., 2016). Furthermore, *Bdnf* promoter IV displays a decrease in acetylation of histone 3 (H3) and histone 4 (H4) in adult restraint stress, and further reduction in acetylation of H3 and H4 is observed from maternal separation. However, these epigenetic changes can be recovered by escitalopram treatment (Seo et al., 2016).

In addition to postnatal maternal separation, which occurs at a very young age, adolescent social stress in mice also causes epigenetic changes to BDNF in adulthood (Xu et al., 2018). Here, *Bdnf* gene expression is downregulated in the medial prefrontal cortex as a result of adolescent social stress, and increased dimethylation of H3 at lysine 9 (*H3K9me2*) downstream of the *Bdnf* IV promoter – his occurs in conjunction with cognitive flexibility in the mice after reaching adulthood. Both epigenetic changes and behavioral changes can be reverted by antidepressant treatment (Xu et al., 2018). The effect of maternal separation on behavior has been examined in a similar study; early-life interaction with a stranger can induce a stressful social experience, and as a result, less social interaction with strangers is observed from pups who have been separated from their mothers (Karen and Rajan, 2019). Furthermore, the stressful social experience subsequently elevated DNA methyltransferase (*Dnmt3a*) as well as other epigenetic elements such as decreased acetylation and increased methylation of histones in the amygdala of rats, which had been raised under maternal separation (Karen and Rajan, 2019).

In addition to stress-induced epigenetic changes, a recent study also investigates the effect of early life stress across generations and gender. By subjecting the first generation of rats to maternal separation and the subsequent generation raised in a balanced cross-fostering manner, it was found that early-life stress through maternal separation resulted in increased *Bdnf* methylation in both male and female rats, but *Bdnf* expression was reduced only in females (Coley et al., 2019). Subsequently, the second-generation rats from an early life social stress lineage exhibited increased *Bdnf* methylation, while fostered female rats raised by a first-generation mother, which had previously undergone early life stress exhibited *Bdnf* methylation (Coley et al., 2019). These studies suggest that stress-induced epigenetic changes are carried across generations.

## Treatments Attenuating Early-Life Social Stress Changes

While early-life social stress may induce adverse epigenetic and behavioral changes, these changes might not be entirely irreversible. As noted above, the use of antidepressants such as escitalopram has proven effective in recovering epigenetic changes in the *Bdnf* gene in both humans and rats (Seo et al., 2016; Wang et al., 2018b). Lithium treatment has been noted to reverse the effects of early-life social stress by increasing neuropeptide Y and corticotropin-releasing hormone – both associated with depression and stress vulnerability – in the adult rat hypothalamus (Husum and Mathé, 2002). Valproic acid treatment helps treat cognitive dysfunction induced by amphetamine to mimic a later-life social stress event. Still, a combination of both early life stress and later life stress renders the treatment ineffective (Pinheiro et al., 2012). Other treatments attenuating early-life social stress changes can be found in corticotropin-releasing hormone blockers, which recover early-life social stress-induced hippocampal dysfunction (Ivy et al., 2010), or the use of dopamine receptor 3 (Drd3) agonists to increase dopaminergic neuronal activity, which has been shown to restore normal social behavior in mice that have undergone early-life social stress (Shin et al., 2018).

## Limitations and Future Perspectives

While the role of epigenetics in regulating 5-HT-associated genes under early-life social stress appears to be backed by substantial evidence, limitations remain when it comes to translating those results to clinical research; after all, the same methods used to directly study the expressions of genes in the rodent brain cannot be effectively implemented with humans. The relative scarcity of neurological data available means that investigation of peripheral expression levels for genes such as 5-HTT (Olsson et al., 2010) and BDNF (Lopez et al., 2013) in association with epigenetic regulation under exposure to early life stress is necessary for future research. Furthermore, future studies may also want to consider and compare the effect of the social and natural environment on epigenetic regulation; stress in early life can come through various means, and as such, natural obstacles such as food deprivation may yet generate different responses compared to social stress.

Even so, epigenetic studies have proven to be highly useful in improving our understanding of the biological processes that serve as the fundament for social influences on health. By combining human epidemiological studies and animal model experimental studies, the role of epigenetic mechanisms in social stress-related health risks should become clearer. This would help advise public health and social interventions, which serve to reduce epigenetic aging and improve long-term health.

## Summary

**Figure 1:**
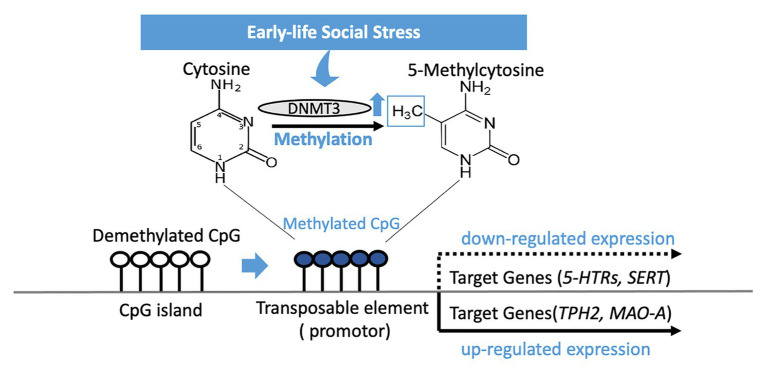
DNA methylation of CpG islands in the genetic code silences expression of a gene after early-life social stress exposure. Methylation occurs by a methyltransferase (DNMT3) transferring a methyl group to cytosine, and when occurring on a transposable element has the effect of repressing gene transcription related to that element. *5-HTRs*, 5-HT receptors; *SERT*, serotonin transporter; *TPH2*, tryptophan hydroxylase 2; *MAO-A*, *monoamine oxidase* A.

Early life social stress may be a driving force for susceptibility to depression in later years, and epigenetic regulation of serotonin-associated genes is another means by which early-life social stress exerts its influence (Figure 1). Genetic changes to the associated genes in later life have proven to be a strong indicator for depressive disorders in both animal models and clinical studies – this suggests that targeted recovery of these epigenetic changes is a potential path to take when considering treatment of the major depressive disorder. Epigenetic changes to serotonin-associated genes are tied directly to increased or decreased genetic expression, which in turn is correlated to behaviors distinctive of depressive disorders. However, one thing of note when it comes to epigenetic changes in the sexual dimorphism present in their effects is an observation that is recurring in both BDNF and MAOA, where the presence of the epigenetic changes might be opposed or non-existent depending on the sex. Regardless, as progress in epigenetics advances, greater understanding and better treatment philosophies for depression may arise in the future.

## Author Contributions

TS and IP designed the flow of this review paper and edited the manuscript. TS and CT wrote the main manuscript and prepared a figure. All authors contributed to the article and approved the submitted version.

### Conflict of Interest

The authors declare that the research was conducted in the absence of any commercial or financial relationships that could be construed as a potential conflict of interest.
